# Tobacco use and its associated factors among middle and old-aged women in India using LASI wave-1 data

**DOI:** 10.1371/journal.pone.0298663

**Published:** 2024-03-05

**Authors:** Manik Halder, Nuruzzaman Kasemi, Malasree Majumder

**Affiliations:** 1 Raiganj University, Raiganj, West Bengal, India; 2 Birpara College, Birpara, West Bengal, India; University of Gour Banga, INDIA

## Abstract

**Background:**

Despite a global decline in tobacco use reported by the Global Adult Tobacco Survey (GATS), India stands out for its high number of tobacco users. While research in India often focuses on tobacco use among young adults, there’s a notable lack of studies addressing tobacco use among middle and old-aged women. However, the national prevalence of tobacco use among middle and old-aged (45 years and above) women in India is 18.2%. Thus, this study seeks to identify the factors influencing tobacco consumption among middle and old-aged women in the country.

**Methods:**

The study has utilized secondary data from Longitudinal Aging Study in India (LASI) conducted in 2017–18. This study exclusively included a total of 38,180 middle and old-aged women who reported tobacco use. The analysis encompassed the utilization of Chi-square tests and Binary logistic regression to pinpoint the risk factors linked to tobacco use among middle and old-aged women.

**Results:**

Our study reveals a heightened likelihood of tobacco use among middle and old-aged women residing in the northeastern region of India. The study underscores the imperative to direct targeted efforts toward middle and old-aged women who fall into specific categories, including those who are widowed, separated, or unmarried, individuals who consume alcohol, those with lower socioeconomic and educational standings, residents of rural areas, those living in solitude, individuals experiencing depressive symptoms, and those who self-report poor health.

**Conclusion:**

Given the heightened susceptibility of these demographic groups to tobacco use, it is crucial to prioritize tobacco prevention and cessation initiatives specifically tailored to their needs and circumstances.

## 1. Introduction

Tobacco use poses a growing global public health challenge that transcends geographical, demographic, and socioeconomic boundaries [1, 2]. Acknowledged by the World Health Organization (WHO) as a significant threat, tobacco use results in over 8 million global deaths annually [3]. Its impact spans across all age groups, from youth to the elderly, influencing not just individual health but also societal well-being and economic stability. Linked to various debilitating diseases such as cardiovascular conditions, cancer, respiratory ailments, and strokes [4–6],

Understanding tobacco use among older women in India is of paramount importance. India’s aging population presents unique challenges as non-communicable diseases become more prevalent with age [7]. Given the profound implications of tobacco use on this aging demographic, exploring its intersection with health challenges, disabilities, and increased healthcare utilization becomes imperative [8–11]. Beyond health concerns, tobacco use among middle and old-aged women in India poses complex social and economic challenges [12]. India’s diverse cultural, socioeconomic, and demographic landscape creates a unique context for investigating tobacco use among this demographic [13, 14]. Socioeconomic and demographic factors such as income, education, marital status, and occupation influence tobacco use patterns, emphasizing the importance of understanding these associations [15–20].

Despite India’s significant efforts to combat tobacco use, research has predominantly focused on the younger population, resulting in middle and old-aged women being understudied [21–23]. Nevertheless, the prevalence of tobacco use among middle and old-aged women in India remains notably high, standing at 18.2% [24]. While the adverse effects of tobacco consumption are well-documented, there is a substantial gap in comprehensive research aimed at understanding tobacco use patterns among older populations, especially among middle and old-aged women in low- and middle-income countries like India [25–27]. This study aims to fill this gap by examining the factors influencing tobacco use among middle-aged and older women in India. It analyzes these factors considering regional differences, offering insights for decision-makers, researchers, and public health professionals. By doing so, the study aspires to contribute to the identification of potential areas for future research and facilitate better preparation for future tobacco control efforts.

## 2. Material and methods

### 2.1 Study design and sample population

**Fig 1:**
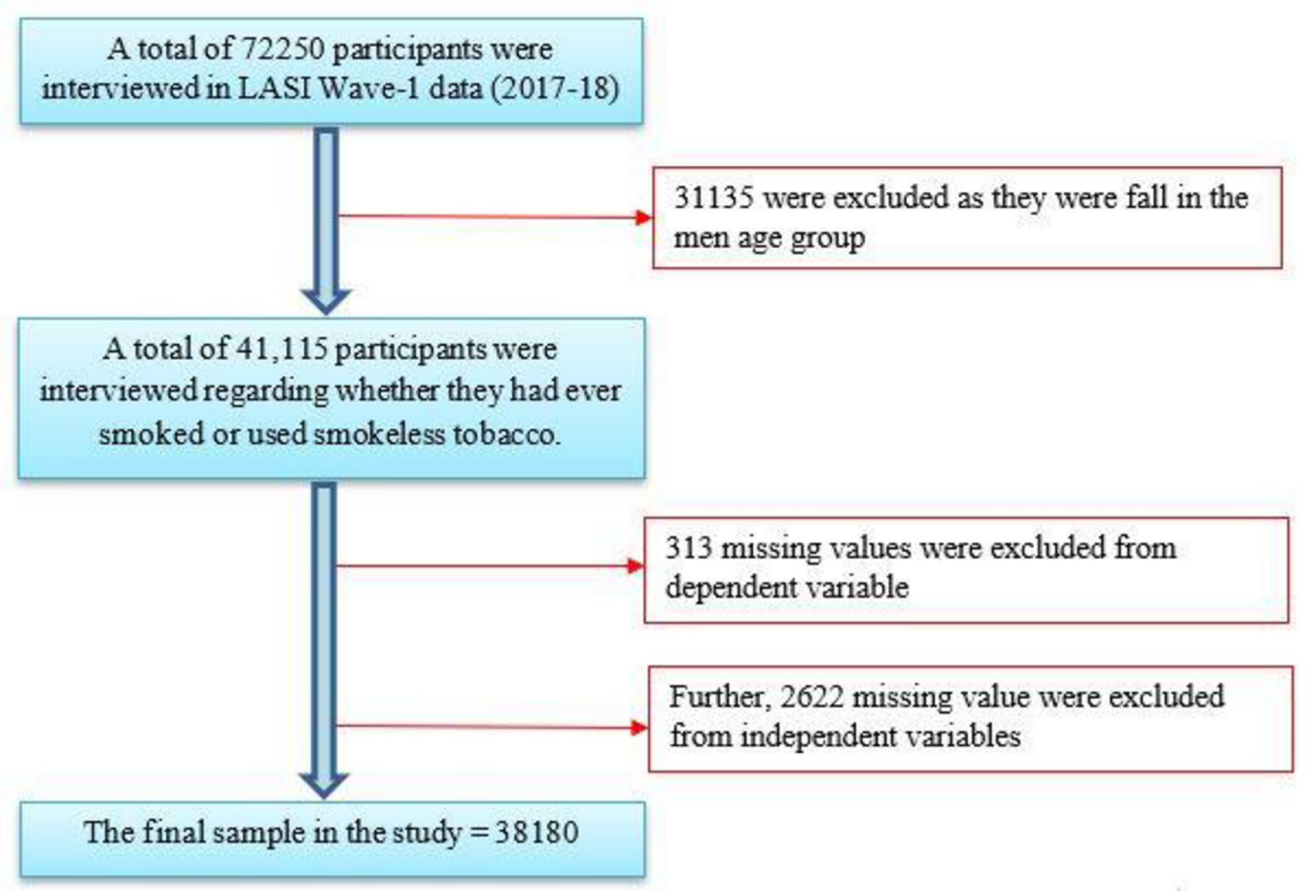
Sample selection procedure.

The Longitudinal Aging Study in India (LASI), published in 2021, provided data for the current study. The LASI is a large survey used to better understand the socioeconomic, demographic, behavioral, and health-related factors that influence the health of milddle and old-aged populations in India. The LASI survey was conducted through a partnership with the International Institute for Population Sciences (IIPS), Havard T. H. Chan School of Public Health (HSPH), and the University of Southern California (USC). In LASI, the study population was selected using a multistage stratified sampling technique. For rural areas, a three-stage sampling strategy was adopted, while for urban areas, a four-stage sampling strategy was adopted. The LASI collected data on 72,250 respondents across all Indian states and union territories except Sikkim. Hence, data from Sikkim could not be included in this analysis. Data collection and quality are maintained and supervised by the International Institute for Population Sciences (IIPS). Out of 72,250 samples, 38,180 effective samples were considered for the present study. The process of sample selection has been presented in Fig 1.

### 2.2 Dependent variable

In this study, the dependent variable under investigation pertains to the extent of tobacco use among middle and old-aged women. To operationalize this variable, responses to the query, "Have you ever smoked or used smokeless tobacco?" were employed. Specifically, respondents answering in the affirmative were assigned a code of "1," while those responding negatively received a code of "0."

### 2.3 Independent variables

To identify the independent variables in our study, we conducted a thorough review of pertinent literature sources [21, 23, 26, 28–30]. We considered a range of variables encompassing socioeconomic, demographic, geographic, behavioral, and health-related aspects that could potentially impact tobacco use among the study’s participants. Within the realm of socioeconomic and demographic factors, we included age, marital status, social group, religion, Monthly Per Capita Expenditure (MPCE) quintile, schooling, pension status, and variables related to prayer. The variable Age is dichotomized into Middle (45–59 years) coded as 1 and Old (60 years and above) coded as 2. Marital status is categorized as " currently in union " (coded as 2) and " currently not in union." (coded as 1). Social group categories include SC (Scheduled Caste, coded as 1), ST (Scheduled Tribe, coded as 2), OBC (Other Backward Classes, coded as 3), and None of Them (coded as 4). Religion categories are classified as Hindu (coded as 1), Muslim (coded as 2), Christian (coded as 3), and Others (coded as 4). MPCE quintile is grouped into Poor (coded as 1), Medium (coded as 2), and Rich (coded as 3). Schooling is dichotomized into Yes (coded as 2) and No (coded as 1). Pension is classified as Yes (coded as 2) and No (coded as 1). Predictors encompass the frequency of religious prayer, including Daily, More than Once a Week, Once a Week, 1 to 3 Times a Month, 1 or More Times a Year, and Never. These factors were deemed relevant as they have been previously associated with variations in tobacco use patterns. Behavioral and health-related aspects were also examined, comprising variables that assess alcohol consumption, depression, self-reported health, activities of daily living (ADL), and instrumental activities of daily living (IADL). Alcohol consumption is categorized as Yes (coded as 2) and No (coded as 1). Depression is classified as Yes (coded as 2) and No (coded as 1). Self-rated health (SRH) is categorized as Good (coded as 2) and Poor (coded as 1). Living arrangements are divided into two categories: Living Alone (coded as 1) and Living with Spouse, Children, and/or Others (coded as 2). ADL and IADL assessments are widely employed in gerontology and healthcare to gauge an individual’s functional status. These evaluations play a pivotal role in comprehending a person’s capacity to carry out essential daily tasks autonomously. In the current study, ADL comprises tasks such as dressing, walking across a room, bathing, managing eating difficulties, and getting in or out of bed. On the other hand, IADL encompasses activities like preparing a hot meal, shopping for groceries, making telephone calls, adhering to medication regimens, performing household or gardening tasks, managing financial matters (including bill payment and expense tracking), and navigating or locating addresses in unfamiliar places [21]. Responses in these assessments are binary, indicating whether the individual can independently perform the task (coded as 1) or requires assistance (coded as 0). These factors were chosen because they reflect various behavioral and health-related dimensions that influence tobacco use among middle and old-aged women.

**Fig 2:**
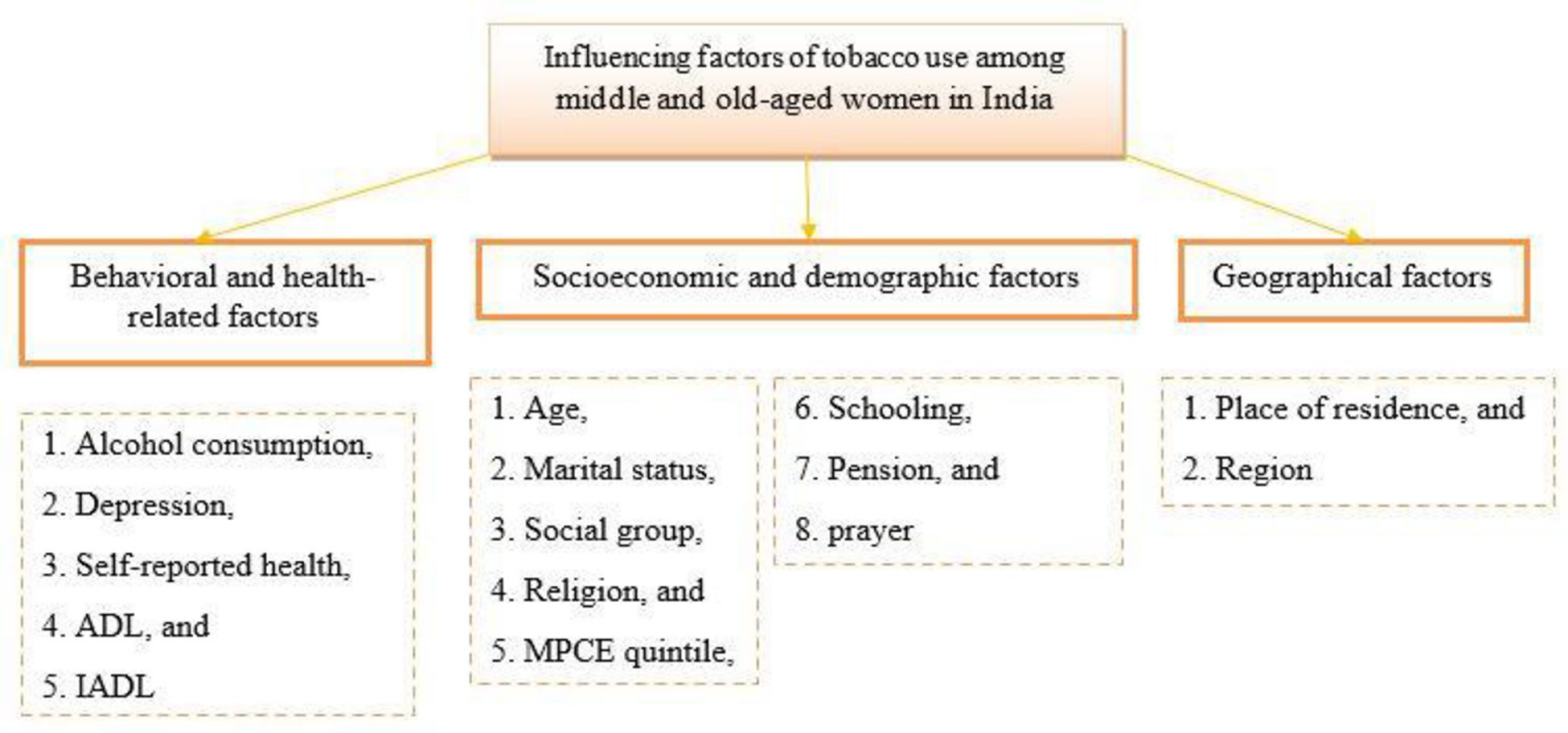
Conceptual framework showing the factors of tobacco use among middle and old-aged women in India, 2017–18.

Additionally, we incorporated geographical factors into our analysis by considering place of residence and regional location. Place of residence is categorized as Rural (coded as 2) and Urban (coded as 1). Geographical regions are divided into North (coded as 1), Central (coded as 2), Eastern (coded as 3), Western (coded as 4), Southern (coded as 5), and Northeastern (coded as 6). This was essential to account for potential regional disparities in tobacco use within India [8]. These independent variables were deliberately chosen to establish a comprehensive analytical framework, as illustrated in Fig 2. The selection aimed to unveil the multifaceted determinants influencing tobacco use among middle and old-aged women in the Indian context.

### 2.4 Statistical approach

All analyses of the study were performed with the help of Stata software. Firstly, the distribution of individuals by socioeconomic variables was determined by descriptive analysis. Then, to determine the prevalence of tobacco use among middle and old-aged women by background characteristics, we used bivariate analysis with the help of Pearson’s chi-square testing. Finally, factors associated with tobacco use were investigated using the Binary logistic regression model. Adjusted odds ratio (AOR) and unadjusted odds ratios (uOR) with 95% confidence intervals (CIs) are presented in Table 2. The model can be specified as

$$\text{Log}=\left(\frac{\pi }{1-\pi }\right)=\beta_{0}+\beta_{1X_{1}}+\beta_{2X_{2}}+\cdots \cdot \beta_{nX_{n}}$$

Where,

π represents the probability of an event (here tobacco use),

*β*_0_ represents the y-intercept,

*β*_1_ displays the regression coefficients related to the control group, and

*x*_1_represents the independent variable

We tested the existence of multicollinearity among the independent variables included in the ordered regression model for the purposes of our study. According to Alkan and Güney, those with a variance inflation factor (VIF) of 5 and above would indicate an intermediate degree of multicollinearity, and those with a VIF of 10 or above would indicate a high level of multicollinearity. This investigation did not find any variables that could cause a multicollinearity problem between the variables. The mean VIF value of our study was 1.41.

## 3. Results

### 3.1 Socioeconomic characteristics of respondents in the study

Descriptive characteristics of middle and old-aged women are shown in Table 1. Most of the respondents (69%) lived in rural areas, 32% were widowed, separated, or divorced, 79% suffered from depression, and 41% of them were 60 years or older. About 46% of OBCs, 83% were Hindus. A significant number of respondents (62% were not attending school, 43% were poor, and 95% lived with a spouse, child, or others), and 18% had poor self-reported health. Religious prayer was regularly practiced by respondents (61%). About 41% of respondents said they had trouble doing IADLs, only 9% had ADLs, and 91% of respondents did not receive a pension.

**Table 1:** Characteristics of respondents and prevalence of tobacco use among middle and-old aged women in India.

Characteristics	Frequency (*n*)	Percentage (%)	Tobacco use		
			Frequency (*n*)	Prevalence (%)	*p*-value
**Age**					<0.001
**Middle age**	22,447	58.8	3,410	15.2	
**Old age**	15,733	41.2	3,585	22.8	
**Social group**					
**SC**	7,745	20.3	1,853	23.9	<0.001
**ST**	3,432	9.0	992	28.9	
**OBC**	17,640	46.2	2,645	15.0	
**None of them**	9,363	24.5	1,505	16.1	
**Religion**					
**Hindu**	31,575	82.7	5,826	18.4	<0.001
**Muslim**	4,224	11.1	833	19.7	
**Christian**	1,053	2.8	187	17.7	
**Other**	1,328	3.5	149	11.2	
**Marital status**					
**Currently in union**	25,873	67.8	4,113	15.9	<0.001
**Currently not in union**	12,307	32.2	2,882	23.4	
**Living arrangements**					
**Alone**	1,796	4.7	487	27.1	<0.001
**Living with spouse, children, and others**	36,384	95.3	6,508	17.9	
**MPCE quintile**					
**Poor**	16,237	42.5	3,340	20.6	<0.001
**Middle**	7,688	20.1	1,458	19.0	
**Rich**	14,256	37.3	2,197	15.4	
**Schooling**					
**Yes**	14,678	38.4	1,565	10.7	<0.001
**No**	23,502	61.6	5,429	23.1	
**Prayer**					
**Daily**	23,239	60.9	4,056	17.5	<0.001
**More than once in a week**	3,868	10.1	575	14.9	
**Once in a week**	3,451	9.0	589	17.1	
**1 to 3 times a month**	2,077	5.4	475	22.8	
**1 or more times in a year**	2,256	5.9	505	22.4	
**Not at all**	3,289	8.6	795	24.2	
**Alcohol consumption**					
**Yes**	965	2.5	551	57.1	<0.001
**No**	37,215	97.5	6,444	17.3	
**Depression**					
**No**	30,186	79.1	5,268	17.5	<0.001
**Yes**	7,994	20.9	1,727	21.6	
**ADL**					
**Yes**	6,138	16.1	1,358	22.1	<0.001
**No**	32,042	83.9	5,637	17.6	
**IADL**					
**Yes**	15,758	41.3	3,420	21.7	<0.001
**No**	22,422	58.7	3,575	15.9	
**Self-rated health**					
**Good**	31,315	82.0	5,491	17.5	<0.001
**Poor**	6,865	18.0	1,504	21.9	
**Pension**					
**Yes**	3,329	8.7	492	14.8	<0.001
**No**	34,851	91.3	6,503	18.7	
**Place of residence**					
**Rural**	26,273	68.8	5,682	21.6	<0.001
**Urban**	11,907	31.2	1,313	11.0	
**Region**					
**North**	4,638	12.1	555	12.0	<0.001
**Central**	7,440	19.5	1,381	18.6	
**East**	8,852	23.2	2,230	25.2	
**North-east**	1,282	3.4	498	38.8	
**West**	6,419	16.8	1,245	19.4	
**South**	9,548	25	1,086	11.4	

### 3.2 Prevalence of tobacco use by different characteristics of respondents in India

In the present study, tobacco use among respondents in India by background characteristics is shown in Table 1. Respondents who were never married, separated, or divorced had a very high prevalence of tobacco use (23%) than their counterparts. Moreover, it was twice as common among respondents living in rural areas than in urban areas (22% vs. 11%). Compared to respondents who did not drink alcohol, the prevalence of tobacco use was nearly 4 times higher (57% vs. 17%). As with their peers, it was also significantly higher among respondents who were in the poorest MPCE quintile (21%) and living alone (27%). Regarding sociocultural characteristics, the prevalence was highest among Muslims (20%) and Scheduled Caste (29%). When it came to religious prayer, respondents who regularly participated in it were significantly less likely to report tobacco use than their counterparts.

**Fig 3:**
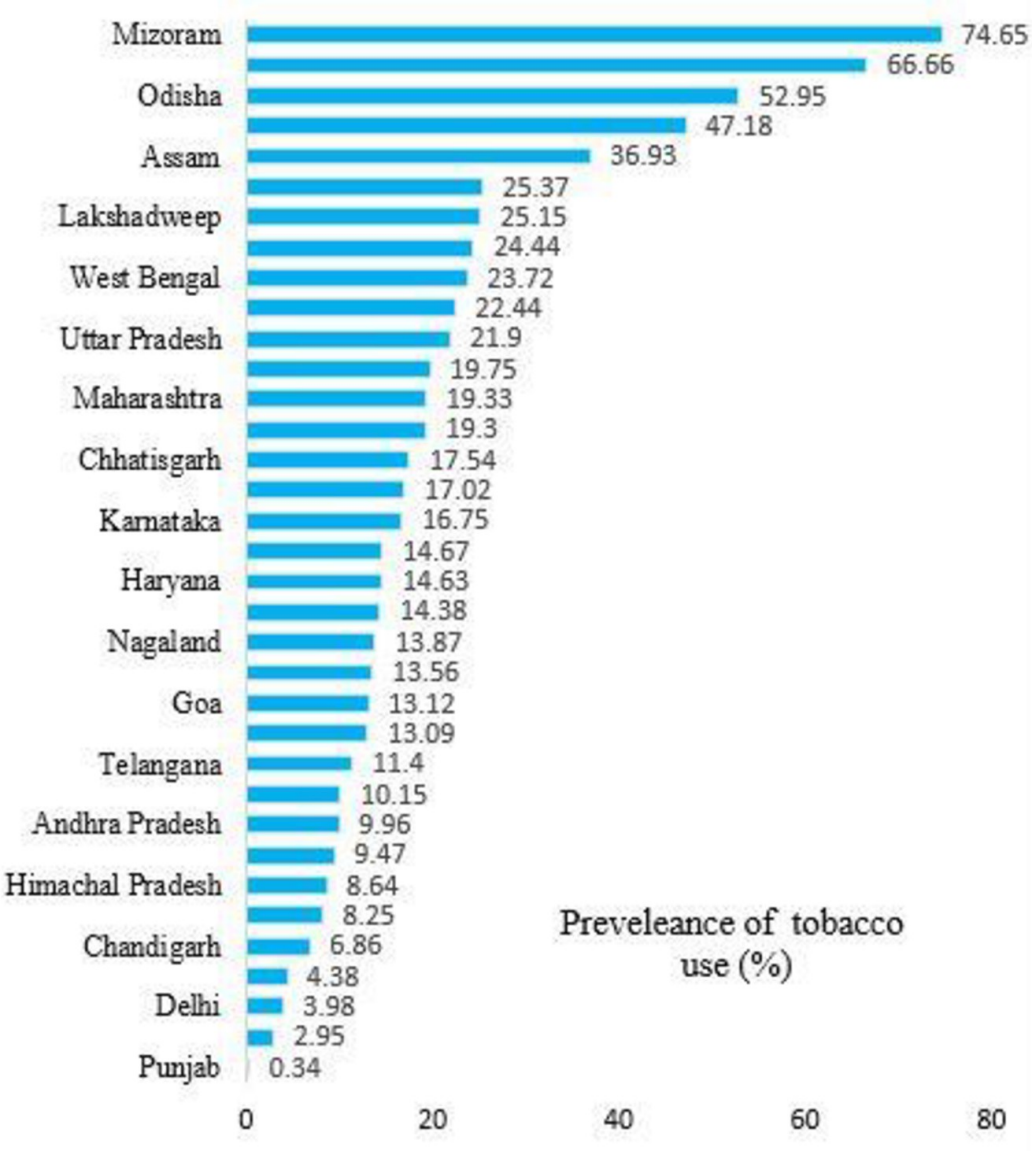
State and union territory wise prevalence of tobacco use among middle and old-aged women in India, 2017–18.

Similarly, those who reported feeling depressed were 22% more likely to use tobacco than those who did not use tobacco (17%). In terms of attendance, respondents who did not attend school had a significantly higher prevalence of tobacco use (23%). A slightly higher prevalence of tobacco use was found among respondents with ADL and IADL limitations (22%, 22%) compared to women without ADL and IADL limitations (18%, 16%). Compared to their counterparts, respondents with poor self-reported health had a higher prevalence of tobacco use (22% vs. 18%). The Northeast region had the highest prevalence of tobacco use (39%), followed by the East (25%) and Central (19%). The prevalence of tobacco use among states of India showed significant variation from state to state, as shown in Fig 3.

### 3.3 Influencing factors of tobacco use among older women in India

In Table 2, the probability of tobacco use differed significantly between places of residence. In contrast to their rural counterparts, respondents in urban areas were 56% less likely to be associated with tobacco use (AOR = 0.658; 95% CI = 0.568–0.762). Socio-demographic factors such as religion, social group, and marital status were found to be significant predictors of tobacco use. The likelihood of tobacco use increases with the age of the respondent (AOR = 1.302; 95% CI = 1.174–1.444). Respondents without schooling were 86% (AOR = 1.861; 95% CI = 1.626–2.130) more likely to use tobacco than respondents with schooling. Similarly, among respondents who were not currently in union (AOR = 1.285; 95% CI = 1.146–1.441), the odds were 28% higher than their counterparts. Among religious groups, Muslims were 38% (AOR = 1.383; 95% CI = 1.157–1.653) more likely to use tobacco than others. Tobacco use was significantly related to social group or caste. social group (OBC, and none of them) use about 30% to 40% less tobacco (AOR = 0.608; 95% CI = 0.536–0.691) (AOR = 0.705; 95% CI = 0.613–0.812) than SC. There was a negative association between tobacco use and religious prayer. Compared to their counterparts, respondents who never or rarely attended religious prayer were shown to have a higher risk of tobacco use than those who attended religious prayer (AOR = 0.725; 95% CI = 0.594–0.885). Similarly, respondents who felt depressed (AOR = 1.217; 95% CI = 1.069–1.387) were 21% more likely to use tobacco than those who never felt depressed. The likelihood of tobacco use was about 80% lower among respondents who never drank alcohol (AOR = 0.222, 95%, CI = 0.181–0.272) than among those who drank alcohol. Respondents with poor self-reported health had a higher risk of tobacco use (AOR = 1.140, 95%, CI = 1.009–1.287) than those with good self-reported health. Additionally, it was investigated whether IADL limitations were a highly significant predictor of tobacco use, and it was more common among respondents who had IADL limitations (AOR = 1.130; 95% CI = 1.018–1.254). Geographical region is strongly associated with the likelihood of tobacco use. The highest odds were seen in the Northeast region (AOR = 5.260; 95% CI = 4.469–6.191), followed by the East (AOR = 2.211; 95% CI = 1.930–2.533), the West (AOR = 1. 1.977; 95% CI = 1.677–2.332) and the Central (AOR = 1.448; 95% CI = 1.246–1.684) regions of India.

**Table 2:** Influencing factors of tobacco use among middle and old-aged women in India, 2017–18.

	Unadjusted Odds Ratio	95% CI	Adjusted Odds Ratio	95% CI
**Age**				
Middle age	1.00		1.00	
Old age	1.648***	(1.503–1.807)	1.302***	(1.174–1.444)
**Social group**				
SC	1.00		1.00	
ST	1.293***	(1.125–1.487)	0.888	(0.755–1.045)
OBC	0.561***	(0.497–0.633)	0.608***	(0.536–0.691)
None of them	0.609***	(0.538–0.689)	0.705***	(0.613–0.812)
**Religion**				
Hindu	1.00		1.00	
Muslim	1.086	(0.894–1.320)	1.383***	(1.157–1.653)
Christian	0.952	(0.795–1.140)	0.528***	(0.426–0.654)
Other	0.560***	(0.415–0.754)	0.486***	(0.358–0.660)
**Marital status**				
Currently in union	1.00		1.00	
Currently not in union	1.618***	(1.468–1.783)	1.285***	(1.146–1.441)
**Living arrangement**				
Alone	1.00		1.00	
Living with spouse, children and others	0.586***	(0.494–0.694)	0.788**	(0.656–0.945)
**MPCE quintile**				
Poor	1.00		1.00	
Middle	0.904*	(0.805–1.016)	1.037	(0.910–1.181)
Rich	0.704***	(0.633–0.782)	0.953	(0.848–1.070)
**Schooling**				
Yes	1.00		1.00	
No	2.516***	(2.261–2.801)	1.861***	(1.626–2.130)
**Prayer**				
Daily	0.826***	(0.716–0.953)	0.966	(0.822–1.134)
More than once in a week	0.972	(0.826–1.145)	0.725***	(0.594–0.885)
Once in a week	1.400***	(1.193–1.644)	0.852	(0.698–1.040)
1 to 3 times a month	1.364***	(1.098–1.695)	1.008	(0.814–1.249)
1 or more times in a year	1.508***	(1.264–1.800)	0.877	(0.705–1.090)
Not at all	1.00		1.00	
**Alcohol consumption**				
Yes	1.00		1.00	
No	0.157***	(0.133–0.186)	0.222***	(0.181–0.272)
**Depression**				
No	1.00		1.00	
Yes	1.303***	(1.166–1.457)	1.217***	(1.069–1.387)
**ADL**				
Yes	1.331***	(1.184–1.496)	0.94	(0.817–1.082)
No	1.00		1.00	
**IADL**				
Yes	1.461***	(1.333–1.602)	1.130**	(1.018–1.254)
No	1.00		1.00	
**Self-rated health**				
Good	1.00		1.00	
Poor	1.319***	(1.193–1.459)	1.140**	(1.009–1.287)
**Pension**				
Yes	1.00		1.00	
No	1.323***	(1.149–1.523)	1.109	(0.950–1.296)
**Place of residence**				
Rural	1.00		1.00	
Urban	0.449***	(0.391–0.517)	0.658***	(0.568–0.762)
**Region**				
North	1.00		1.00	
Central	1.676***	(1.462–1.921)	1.448***	(1.246–1.684)
East	2.477***	(2.190–2.803)	2.211***	(1.930–2.533)
North-east	4.670***	(4.083–5.343)	5.260***	(4.469–6.191)
West	1.770***	(1.527–2.052)	1.977***	(1.677–2.332)
South	0.944	(0.784–1.136)	1.064	(0.871–1.300)

*** if p < 0.01,

** if p < 0.05,

* if p < 0.1. CI = confidence interval, ® = reference category.

## 4. Discussion

The results of the Binary logistic regression analysis of the present study showed that the prevalence of tobacco use among middle and old-aged women in India varied dramatically by socioeconomic, demographic, geographic, behavioral, and health-related factors. Older women are more likely to use tobacco than middle-aged women in India. According to the GATS data, it is clear that tobacco use increases with age [12]. This may be because tobacco is more widely adopted when a certain age is reached. A similar result has been found in this study.

Similar to the present study, many previous studies have shown that middle and old-aged SC/ST women consume significantly more tobacco than middle and old-aged women of other castes, suggesting that the practice is widely accepted in society [31, 32]. The present study also indicated that an individual’s attendance at school is a significant predictor of the likelihood of exposure to tobacco. Tobacco use has been found to be positively related to a lack of awareness, social practices, and socioeconomic backwardness, as previously suggested in other studies [33, 34]. Both cognitive abilities and information about health are improved through schooling. Consequently, women’s education can improve their ability to manage the costs associated with poor health, and education can improve knowledge about the negative effects of tobacco use [35].

Similarly, due to their religious prohibition against alcohol consumption, Muslims are more likely to use tobacco than Hindus, Christians, or any other religious group identified in the study. The study discovered that middle and old-aged women who engage in regular religious prayers are more inclined to maintain an ideal lifestyle and demonstrate a tendency to refrain from tobacco use [36].

In developing countries, women’s marital status is a risk factor for tobacco use. A key finding of the study was that middle and old-aged women who were single, divorced, separated, or widowed were more likely to use tobacco than middle and old-aged women who were still married [37]. The living arrangement that is affected by the absence of emotional and social support is significantly associated with tobacco use. Studies have shown that middle and old-aged women living alone are more likely to use tobacco than middle and old-aged women living with spouses, children, and others [38, 39]. Depression is brought on by isolation and a lack of social, emotional, and financial support. In our investigation, we found a link between tobacco use and depression. Tobacco use is higher among depressed middle and old-aged women [40]. Health status, which is also associated with social support, economic support, and depression, is an important predictor of tobacco use. According to the present study, middle and old-aged women with poor health are slightly more likely to use tobacco than those in good health. Similar results have been found in other studies [41–43]. Another finding of the study showed that a significant proportion of middle and old-aged women with greater functional capacity (ADL and IADL) reported higher tobacco use, which is consistent with previous studies in India [31]. The study also revealed a positive relationship between pension and tobacco exposure. In other words, middle and old-aged women who receive a monthly pension are more likely to be exposed to tobacco use than middle and old-aged women who do not receive the pension. Numerous studies have found similar results [44, 45]. According to the findings, women who drink alcohol are more likely to be exposed to tobacco use than non-drinking women. These results are consistent with those found in other investigations [31, 46, 47].

As in other studies, this present study found that the prevalence of tobacco use among middle and old-aged women is higher in rural areas than in urban areas [48, 49]. Middle and old-aged women in India prefer tobacco products as it is more widely available and at lower prices. Lack of information about the harmful effects of tobacco use among rural women residents contributes to their tobacco use behavior. Even after accounting for individual-level socioeconomic and demographic factors, the likelihood of tobacco use varies dramatically across regions in India [27]. Geographically, tobacco use rates are higher among middle and old-aged women in the Northeast, East, Central, and Western regions of India, especially Mizoram, Tripura, Assam, Manipur, Meghalaya, Odisha, Bihar, and West Bengal. The differences reflect regional sociocultural patterns as well as the impact of different government policies on tobacco in different regions. Future research should investigate the reasons for these differences in the regions to better understand how different government policies affect tobacco use and how these policies interact with local sociocultural trends.

### 4.1 Policies and recommendations

To control the manufacture, supply, distribution, and trade of tobacco products, the Indian government introduced the Cigarettes and Other Tobacco Products Act in 2003. After that, The National Tobacco Control Programme (NTCP) was launched in 2007–2008. Establishing and improving cessation facilities at the district level is a key focus area for the NTCP. A three-tier structure is used to implement the NTCP. These are- the National Tobacco Control Cell at the central level, the State Tobacco Control Cell at the state level, and the District Tobacco Control Cell at the District level. Additionally, it is possible to establish tobacco prevention services at the district level [9].

Despite the passage of more than 15 years since then, tobacco use has persisted as a serious problem as a result of the ineffective implementation and communication of laws and policies. The paper also makes several policy recommendations [50]. First, there is a need to spread awareness of the harm that tobacco use causes on a larger scale. Tobacco control obstacles can be overcome, and the burden associated with tobacco use can be reduced by integrating tobacco cessation programs with health and development programs. The fight against tobacco use cessation may benefit from efficient education campaigns that improve public understanding of the health dangers connected with tobacco use [51].

### 4.2 Strengths and limitations of the study

The strength of this study is that the study looked at tobacco use prevalence and contributing factors, as well as patterns and tobacco cessation among middle and old-aged women. This analysis is complete in light of the entire scenario presented. The results of this study will help policymakers to choose target groups for tobacco exposure during policy initiatives. However, the present study also has some limitations. First, recall bias may affect the results because the prevalence of tobacco use is based on self-reported data. Second, we cannot determine a causal relationship between the independent and dependent variables because the study data are cross-sectional. The analysis may be biased by omitted variable bias because the study employed only variables present in the dataset.

## 5. Conclusion

The identified key influencing factors of tobacco consumption among middle and old-aged women in India indicate a lack of awareness regarding the negative health impacts of tobacco use. This study emphasizes the imperative to target older women in rural areas who are widowed, separated, or unmarried, possess low socioeconomic and educational status, live alone, experience depression, engage in alcohol consumption, and exhibit poor overall health. Within this demographic, the ready accessibility of both smoke and smokeless tobacco products is a concerning factor. Initiatives aimed at preventing and ceasing tobacco smoking should prioritize these specific population groups, given their heightened susceptibility to tobacco use.

Consequently, it becomes crucial to consider factors influencing tobacco exposure in order to develop effective measures for reducing tobacco use. Policies that raise awareness of these interventions and enhance the accessibility of cessation counseling are still required to diminish tobacco use in India. To address this issue comprehensively, public health strategies should be implemented to increase awareness of the harmful effects of tobacco use on health at the grassroots level.
